# CCL2 and IL18 expressions may associate with the anti-proliferative effect of noncontact electro capacitive cancer therapy *in vivo*

**DOI:** 10.12688/f1000research.20727.2

**Published:** 2020-07-23

**Authors:** Rarastoeti Pratiwi, Nyoman Yudi Antara, Lalu Gunawan Fadliansyah, Syamsul Arif Ardiansyah, Luthfi Nurhidayat, Eti Nurwening Sholikhah, Sunarti Sunarti, Sitarina Widyarini, Ahmad Ghitha Fadhlurrahman, Hindana Fatmasari, Woro Anindito Sri Tunjung, Sofia Mubarika Haryana, Firman Alamsyah, Warsito Purwo Taruno

**Affiliations:** 1 Faculty of Biology, Universitas Gadjah Mada, Yogyakarta, 55281, Indonesia; 2 Graduate School of Biotechnology, Universitas Gadjah Mada, Yogyakarta, 55281, Indonesia; 3 Faculty of Medicine, Public Health, and Nursing, Universitas Gadjah Mada, Yogyakarta, 55281, Indonesia; 4 Faculty of Veterinary Medicine, Universitas Gadjah Mada, Yogyakarta, 55281, Indonesia; 5 Center for Medical Physics and Cancer Research, Ctech Labs Edwar Technology, Tangerang, 15320, Indonesia

**Keywords:** ECCT, rat breast tumor, anti-proliferative, IL18, CCL2

## Abstract

**Background:** Noncontact Electro Capacitive Cancer Therapy (ECCT) is a novel treatment modality in cancer. Chemokine (C-C motif) ligand 2 (CCL2) has a major role in the outgrowth of metastatic breast cancer. Interleukin 18 (IL18) plays a role in macrophage alteration, which leads to excessive angiogenesis. This study aims to elaborate on the association of CCL2, IL18, IL23α, and TNF-α (tumor necrosis factor-alpha) expression with the anti-proliferative effect of ECCT in rat breast tumor tissue.

**Methods:** Low intensity (18 Vpp) and intermediate frequency (150 kHz) alternating current-electric field (AC-EF) between two capacitive electrodes were exposed as external EF to a rat cage. Twenty-four rats were divided into four groups of six replicates. Breast tumor tissues were collected from 7, 12-dimethylbenz[a]anthracene (DMBA)-induced rats. Two groups were non DMBA-induced rats without ECCT exposure (NINT) and with (NIT). The other two groups were DMBA-induced rats without ECCT exposure (INT) and with (IT). Mammary glands and breast tumor tissues were collected from each group and preserved. Hematoxylin-eosin and immunohistochemistry staining were performed on paraffin sections of tissues using anti-PCNA, anti-ErbB2, anti-Caspase3, and anti-CD68. CCL2, IL18, IL23α, and TNF-α mRNA relative expressions were analyzed using qRT-PCR.

**Results:** ECCT exposure may cause the reduction of PCNA protein expression as well as ErbB2 on breast tumor tissues, but it causes the increase of Caspase3 and macrophage CD68 protein. In rat breast tumor tissues of IT groups, the mRNA expression of CCL2 and IL18 are significantly down-regulated, in contrast with the up-regulated expression of these cytokines in tumor tissues of the INT group. IL23α and TNF- α expression remained similar in both groups.

**Conclusion:** CCL2 and IL18 expressions have an association with the inhibition of breast tumor cell proliferation affected by ECCT exposure

## Introduction

Electro electro-capacitive Therapy (ECCT) has been developed as a noncontact alternating current (AC) electric field (EF)-based cancer therapy method. A previous study ^1^ reported that ECCT low intensity (18 peak-to-peak voltage) intermediate frequency (100 kHz) treatment inhibits the growth of MCF-7 cells. Furthermore, this EF therapy could reduce the tumor size of C3H mice-breast tumor model, without abnormality in the dermal tissue and mammary glands of sham mice ^1^. Moreover, Mujib *et al*. ^2^ suggested that ECCT exposure (100–200 kHz) might induce p53 expression in cancer cells, such as oral squamous cell carcinoma, HeLa, and bone marrow mesenchymal cells. A previous study, using Tumor Treating Electric Field (TTFields) demonstrated that AC-EF with low intensity and intermediate frequency exposure to tumor cells caused the failure of tumor cell division toward the mitotic phase ^3, 4^. A following study reported that the failure of tumor cell division was due to the disruption of spindle microtubule assembly, but not in normal cells ^5^. From those studies, it was suggested that the failure of tumor cell division activates tumor cell apoptosis. However, the evidence that AC-EF exposure disturbs cancer cell proliferation and the molecular mechanism underlining this cell disturbance and elimination remains unknown.

Solid tumor growth and development are dependent on inflammatory cells in its microenvironment. Stromal components, such as endothelial cells, myeloid derivate suppressor cells, and macrophages, reside in the solid tumor microenvironment ^6^. Macrophages act as inflammatory cells that can be affected by chemical signals, i.e. cytokines and chemokines ^7, 8^, and electric fields ^9^. A previous study reported that CCL2 is a chemo-attractant that binds to its receptor (CCR2) on monocytes, macrophages, and lymphocytes. In a previous *in vivo* study, CCL2-induced chemokine cascade in macrophage-associated metastasis (MAM) produced another ligand, CCL3, for metastatic seeding of breast cancer cells ^7, 10^ In addition, IL18 plays a role in the migration of breast cancer cells via down-regulation of claudin12 and p38 MAPK (mitogen activation kinase) pathway ^11^. Hoare *et al.* ^9^ demonstrated that electrical signals have been identified as major contributors to the coordination and regulation of macrophage functions. So far, electric fields-based therapies need to be described in order to understand the modulation of macrophage function, which underline the solid tumor microenvironment. The mechanism needs to be investigated.

Tumor necrosis factor-alpha (TNF)-α cytokine is one member of the tumor necrosis factor superfamily with a wide spectrum of biological activity. Meneggati *et al.* ^12^ reviewed TNF-α early on, and this inflammatory cytokine was suggested to be a potential antitumor agent and inducer of apoptosis *in vitro*. However, in the following years, recent studies reported that TNF-α significantly induces breast cancer metastasis via TNFα-activated mesenchymal stem cells (MSCs) in a lung metastasis model of murine breast cancer ^13– 15^. However, under TNF-α stimulation at *in vitro* model, the MSCs can also release IL-10 as anti-inflammatory cytokine that may be a beneficial for cancer growth inhibition ^16^. Although many studies focus on the function of TNF-α in the solid tumor microenvironment, the function remains as yet not fully clarified. So far, we understand that macrophages are multifunctional in the solid tumor microenvironment. Tumor-associated macrophages (TAMs) help tumor cell growth by releasing several pro-inflammatory cytokines, such as TNFα and IL23 ^17^. A previous study suggested that IL-23 is involved in inflammation and angiogenesis activities in the tumor microenvironment in spite of moderating CD8 ^+^ T-cell infiltration ^18^. However, recent study suggested that TAM is an activated M2 macrophage ^19^ Furthermore, the evaluation of IL-23 suggested that this cytokine has a function in promoting tumor metastasis and growth by upregulating matrix metalloproteinase (MMP)-9 ^20^. On the other hand, Zimolag *et al.* ^21^ reported that direct current (DC)-EF in the physiological condition might reposition MCSs into a wound site and allow macrophages to be at a short distance to the wound. Therefore, the expression of either TNFα or IL-23 cytokines-produced inflammatory cells in the microenvironment of solid breast tumor during the physiological DC-EF or AC-EF need to be further examined.

The anti-proliferative effect of AC-EF has been reported using several cell lines and in some tumor animal models. As reported by Ma *et al.* ^22^, DMBA-induced breast cancer enhanced chromosomal instability and increased ErbB2-mediated mammary carcinogenesis. However, the association and mechanism of killing tumor cells and how the immune system clears death cells needs to be further clarified. A recent study demonstrated that TTFields therapy activates macrophage specific immune responses through the activation of several cytokines, such as TNF-α, and IL1-β *in vitro* ^10^. In the present study, we elaborate on the relationship between the anti-proliferative effect of ECCT on DMBA induced-rat breast cancer cell growth and on the activity of inflammatory cells to express cytokines IL18, TNF-a, IL23 and chemokine CCL2, which play an essential role to the development of solid breast tumors. The use of this breast tumor animal model in this study is needed, because this model can represent the condition of breast cancer in patients. The main objective of this study is to examine the effectiveness of ECCT treatment on tumor growth inhibition through the molecular communication among stromal cells in the solid microenvironment tumor.

## Methods

### Animals

This study was carried out at the animal house of LPPT Research Center Universitas Gadjah Mada (UGM) which is accredited by ISO/IEC 17025:2000 (a laboratory management standard). All requirements for animal welfare following the LPPT Ethics Committee Guidance have been fulfilled. This animal experiment has been legalized with an Ethical Clearance certificate number: 00029/04/LPPT/2018. The rat number for this experimental design was calculated for the minimal biological replication of rats (n=6), with four treatment groups according to the Federer Formula ^23^. 24 female rats ( *Rattus norvegicus* Berkenhout, 1769) Sprague Dawley (SD) strain, five weeks old and weighing 50–80 grams were used in this study. The rats ware obtained from LPPT Research Center.

Rats were fed with AIN-93M standard diet and standard water *ad libitum*. Rats were placed in a standard animal room (temperature and humidity were 20 to 25 °C and 40 to 60%, respectively). Rats were acclimatized to the laboratory condition and standard cage for 5 days.

This study focused on samples of rat breast tumor and healthy mammary gland. Therefore, we only observed the minimal number of tissue samples required for replication; three tissues samples (n=3) per treatment group (4 groups) was used for biological replication. Each sample was measured twice for qRT-PCR analysis and triple sections per tissue sample for histopathologic scoring requirements. Each rat was marked individually using picric acid (non-toxic) staining. Rats were observed for behavior, general physical conditions such as hairs, eyes, noose, ears, and feces every day. Rats were weighed every three days during the experiments using the balance scale for rodents. Rat welfare was maintained following the standard protocol from LPPT Research Center.

The experimental design of ECCT exposure treatments used four rat groups, six biological replicates, which consisted of:

- NINT group: non-DMBA-induced rats, non-EF therapy exposure
- NIT group: non-DMBA-induced rats, EF therapy exposure
- INT: DMBA-induced rats, non-EF therapy treatment
- IT: DMBA-induced rate, EF therapy exposure

Rats were induced ten times with DMBA ^24, 25^ doses of 20 mg/kg body weight within five weeks by oral administration. The DMBA (Sigma Aldrich; cat. no. D3254-1G) administrations were done around 04 p.m. in the animal room by the technician of LPPT Research Center following the Standard Operational Procedure (SOP) for DMBA treatment animal. After DMBA administration, all rats were palpated every two days using the standard procedure of palpation from LPPT Research Center. The tumor nodule was observed around 4 to 6 weeks after DMBA administration. Tumor nodule diameters were measured every 2 days using a digital caliper (Fisher Scientific) and all data measurements were tabulated.

Solid tumor (± 1 cm tumor size)-bearing rats were exposed to an AC-EF of 150 kHz and low intensity of 18 Vpp. EF therapy was performed for 21 days, with a total exposure of 10 hours per day with 2 hours rest after first 5 hours exposure. The starting time of ECCT treatments were at 06 to 11 a.m., then at 01 to 06 p.m. During ECCT-exposing in the individual ECCT cage (designed by Ctech Labs Edwar Teknologi, IDN Patent REG. P00201200011), rats were fed with a standard diet and cucumber *ad libitum*, however during rest hours, rats were fed with a standard diet and water *ad libitum* in a communal cage with standard bedding and feeding for 5 rats. The ECCT treatment was finished after 21 days of treatment. Rats were sacrificed (euthanasia) by ketamine hydrochloride (KETALAR ^®^ Pfizer; cat. no. 629-24006) injection with a dosage of 150mg/kg body weight on the day after the last treatment. Rats were sacrificed starting at around 08 a.m. with the standard ethics procedure for rat euthanasia and surgery. After taking the samples, the remaining dead rat bodies were put in the freezer prior to eradication of the carcinogenic (DMBA) contaminated animals using the SOP of the LPPT animal house. Mammary glands and solid tumor tissues were sliced and fixed in 10% NBF (neutral buffer formalin, Bio-Optica; cat. no. 05-K01004) with ratio 5:1 for histological examination and in RNAlater ^®^ (Invitrogen; cat. no. AM7024) solution for total RNA extraction.

### Histological examination

Mammary glands and solid tumor tissues were fixed in NBF and then processed using the paraffin method and stained with hematoxylin-eosin using the procedures provided by Bancroft and Cook ^26^. Summarily, the samples were periodically washed with 70% alcohol and subsequently dehydrated using a higher concentration of alcohol (80–100%). The dehydrated samples were then cleared with toluene (Merck; cat. no. 1083252500) overnight. The samples were infiltrated with paraffin (Merck; cat. no. 1073372500) in a 65°C oven and then embedded with freshly prepared paraffin. The sample paraffin blocks were sectioned with a microtome (Microm HM 315) providing a 4–6 um thick slice, which were then placed on a slide. Later on, the samples were then deparaffinized using xylene (Merck; cat. no. 1086612500), rehydrated using a downgraded concentration of alcohol (96–40%), and finally stained with Hematoxylin (made from Hematoxylin Krist C.I.75290, Merck; cat. no. 1159380025, using Erlich’s formulation) and Eosin solution (made from Eosin Y, CI. 45380, Merck; cat. no. 1159350025). The stained samples were subsequently dehydrated using an upgraded level of alcohol, cleared in xylene, and lastly, mounted with Entellan (Merck; cat. no. 1079600500) and coverslip. The random 50 fields of view on IHC slides of each treatment were observed under Leica ICC50 E at 0.5 µm/pixel resolution

### Immunohistochemistry

The 4–6 um thick paraffin section of samples were placed on a Poly-L-lysine coated slide. The INT and IT tumor tissue samples were then processed using the Starr Trek Universal-HRP Detection Kit (Biocare Medical; cat.no BRR 700 AH, AL10) using the manufacturer’s protocols. In brief, the samples were deparaffinized using xylene and then rehydrated with down-graded concentration of alcohol. The samples were heated in the microwave with sodium citrate buffer pH 6.0 for antigen retrieval for 15 minutes at 95 °C. The samples were soaked with 3% H _2_O _2_ (Sigma-Aldrich) in PBS for 5 min to block endogenous peroxidase and subsequently treated with Background Sniper for 20 minutes for suppressing nonspecific binding. Afterwards, samples were separately incubated with anti-PCNA (ABCAM; cat.no. ab18197), anti-caspase-3 (ABCAM; cat.no. ab13847), anti-CD68 (ABCAM; cat.no. ab201340), and anti-ErbB 2 (ABCAM; cat.no. ab16901) antibodies overnight at 4 °C, followed by Trekkie Universal Link incubation for 60 minutes. Then, the samples were incubated with Trek-avidin HRP Label for staining development and then counterstained with hematoxylin. Lastly, the samples were dehydrated using an upgraded concentration of alcohol, cleared with xylene, and mounted with Entellan and coverslip. Immunohistochemistry slides were observed under Leica ICC50 E at 0.5 µm/pixel resolution with 50 fields of view.

### Quantitative-RT-PCR

Quantitative-RT-PCR (qRT-PCR) was applied for measuring the transcriptomic expression (mRNA) of IL18, CCL2, TNF-α, and IL23α (due to the use of sub unit p19 fragment for primer construction) genes. Isolation of RNA was performed using Total RNA Mini Kit (Blood/cultured cell; Geneaid; cat.no RB100), and cDNA synthesis with Superscript ^®^ III first-strand synthesis supermix (Invitrogen; cat.no 18080-400). Primers were as follows:

- IL23α NM_130410.2 F: CAGGTTCCCATGGCTACAGT, R: TCTGGGGTTTGTTGCTTTTC
- IL18 ^27^ F: CAGACCACTTTGGCAGACTTCA, R: ACACAGGCGGGTTTCTTTTGT
- TNF-α ^27, 25^ F: AGCATGATCCGAGATGTGGAA, R: AATGAGAAGAGGCTGAGGCACA
- CCL2 ^28^ F: 5’ GTGCTGTCTCAGCCAGATGCAGTT 3’, R: 5’ AGTTCTCCAGCCGACTCATTGGG 3’.
- GADPH ^29^ F: 5’ TGACAACTTTGGCATCGTGG 3’, R: 5’ GGGCCATCCACAGTCTTCTG 3’

RT-PCR analysis was performed using Universal SYBR ^®^ Green Supermix paint SsoAdvanced (BIO-RAD; kit. No. 172-5270). The thermal cycling conditions for amplification of IL18, TNFα, IL23α, CCL2 and GADPH were the same for pre-denaturation at 95°C, 30”; and denaturation at 95°C, 10”. However, the annealing conditions were different: IL18 TNFα, and GADPH, 60°C for 10”; IL23, 60°C for 15”; and CCL2, 65°C for 10”. All RT-PCR reactions were done using a BIO-RAD CFX96 ^TM^ Real-Time System, C1000 Touch ^®^Thermal Cycler machine. qRT-PCR data was calculated using the Livak method ^30^.

### Statistical analysis

 Data analysis for IHC was scored automatically using color deconvolution and computerized pixel profiling by IHC Profiler plugin on ImageJ version 1.51 software ^31^

The independent-T test with IBM SPSS Statistics v22 was used for image scoring between groups and for qPCR data analysis. All graphs in this article were created by GraphPrism 7 software.

## Results

### Solid tumor growth

**Figure 1.**
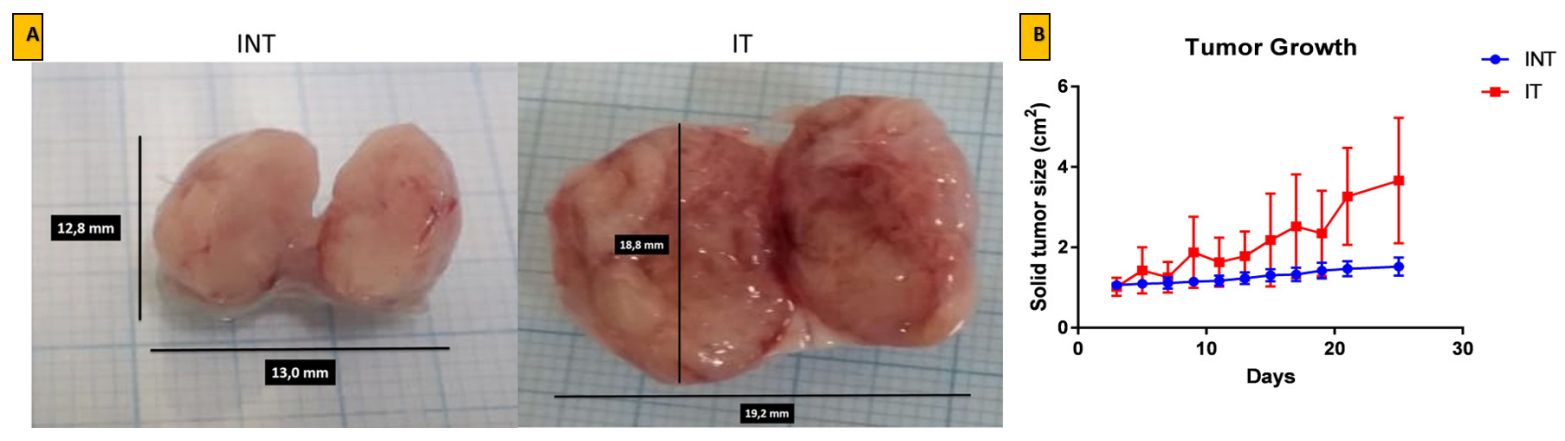
Growth of solid breast cancer mass after ECCT exposure treatment. ( **A**) Representation of solid tumor mass after surgery and ( **B**) the solid breast tumor growth curve based on the diameter of tumor nodules. Tumor growth measurements were taken every two days for 21 days. INT= DMBA-induced rats without EF therapy, IT= DMBA-induced rats with EF therapy exposure.

The rats prior and during ECCT treatment did not have pathogen infection, as observed during daily observation. During the experiments, the rats’ body weight for all groups was not significantly different with the rat base line or untreated rats (secondary data not shown). Results of ECCT-exposed rat breast tumor (IT) show that the increase of the fluid bathing the tumor may affect the tumors size. This is in contrast with the results from the sham rat breast tumor group (INT), which exhibited denser tissues inside the solid tumor and slower growth ( Figure 1).

### Histopathological examination

**Figure 2.**
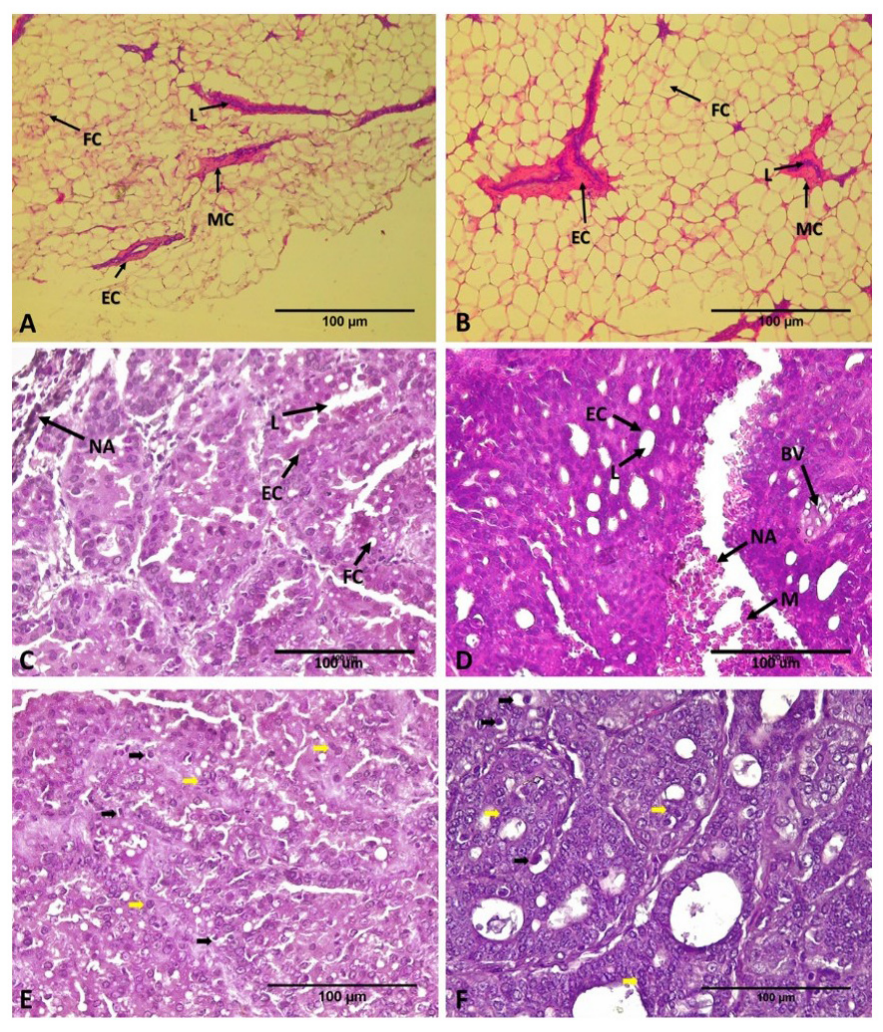
Histological sections of the mammary gland and DMBA-induced rat breast adenocarcinoma after treatment with ECCT exposure. ( **A** and **B**) There is no morphological change both on mammary gland tissue of none DMBA-induced rat non-EF therapy (NINT; **A**), and with EF therapy (NIT; **B**). Hematoxylin and eosin staining, magnification 100x. ( **C** and **D**) In contrast, adenocarcinoma breast tissue of DMBA induced rat non-EF therapy (INT; **C**) shows more massive tumor cells and fewer lumens than the tumor section with EF therapy (IT; **D**). Hematoxylin and eosin staining, magnification 400x. ( **E** and **F**) Mitotic figure (black arrow) and apoptotic figure (yellow arrow) on breast tumor tissues of INT and IT group, respectively. FC= Fat Cells, L=Lumens, EC=Epithelial Cells, M= Myoepithelial Cell, NA= Necrotic Area, BV= Blood Vessels.

Based on histological sections using hematoxylin-eosin staining ( Figure 2), the observation of ECCT exposure effect on average rat mammary glands shows that there is no morphological tissue damage in both treatments, NINT nor NIT rat groups ( Figure 2A and B). In solid tumor tissue sections, DMBA-induced tumor cells grew massively in both treatments ( Figure 2C, D, E and F). Decreasing of fat and myoepithelial cells, and increasing of necrotic cells in mammary tissues due to the high tumor cell proliferation activity and minimal blood supply for healthy cells ^32^. The lumens of mammary glands were filled with massive proliferative cells ( Figure 2 C, D, E and F), in contrast with the healthy mammary gland which contain a layer of epithelial cells ( Figure 2 A and B). According to Denisov *et al*. ^33^, breast tumors develop morphological diversity related to the tumor progression. The morphological tumor cell growth pattern, such as solid tumor, reveals tens to hundreds of groups of shapeless tumor cells. This solid tumor cell pattern is related to tumor invasion or bad prognosis. The tubular structures or tube-shaped pattern of tumor growth is more similar with normal tubular mammary ducts ^33^. Figure 2 C and E (INT group) demonstrated a more solid tumor and less tubular tumor pattern. In contrast, the tubular tumor structure was observed more frequently in the IT group (Figure D and F) than in INT group. In general, tumor growth patterns of both INT and IT groups have solid tumor morphology. Therefore, those tumor can be identified as breast adenocarcinoma ^33^. The breast tumor cells grow invasively to other healthy tissues and caused necrosis area on both treatments, since the healthy myoepithelial and endothelial cells of blood vessels were sited on the solid tumor sections. However, other tissues were removed from the observed-tumor tissue. On the sections of ECCT-treatment (IT), the invasive tumor cells showed a reduced number of mitotic cells and more apoptotic cells ( Figure 2E and F). Moreover, it can be seen that lumen epithelial cells were more frequent for ECCT-exposure treatment than in tumor tissues without ECCT-exposure.

### Immunohistochemistry quantification of protein expression on tumor tissues

**Figure 3.**
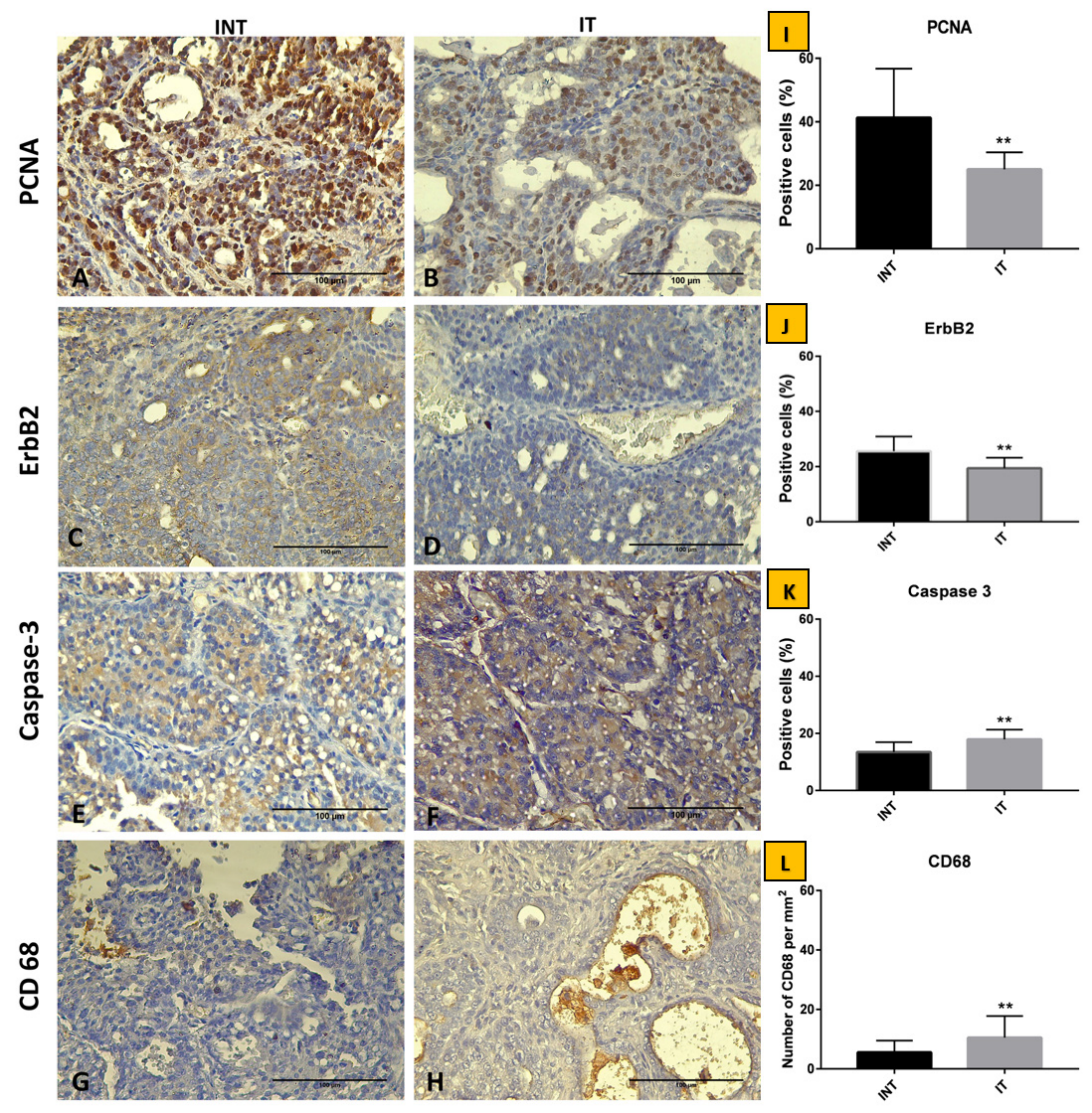
Immunostaining of breast adenocarcinoma tissue sections after ECCT treatment. ( **A** and **B**) Anti-PCNA, ( **C** and **D**) anti-ErbB2, ( **E** and **F**) anti-Caspase-3, ( **G** and **H**) anti-CD68. Percentage of positive cells of ( **I**) PCNA, ( **J**) ErbB2, and ( **K**) Caspase-3 in 50 fields of view. ( **L**) Count of total macrophages in 50 fields of view. Observation of histological slide was performed using Leica CC50 E at 0.5 µm/pixel resolution at 400x. Bar=100 µm. The mean, standard deviation of the data experiment show *, p<0.05, **, p<0.01. INT= DMBA-induced rats without EF therapy, IT= DMBA-induced rats with EF therapy exposure.

Figure 3 shows the appearance of the protein of PCNA, ErbB2, Caspase 3, and macrophage CD68 on solid tumor tissues of ECCT treatment either with ECCT (IT) or without (INT). The percentage of tumor cells expressing PCNA protein in the IT group was significantly lower than in the INT group (p<0.01). Consistent with PCNA expression, ERBB2 protein expression shows a significant decrease in tumor tissues of the IT group compared to the INT group (p<0.05). In contrast, the appearance of Caspase 3 and CD68 proteins on tumor tissues of the IT group was significantly higher than the INT group (p<0.01).

### Relative mRNA expression of CCL2, IL18, TNF-α, and IL23α genes

**Figure 4.**
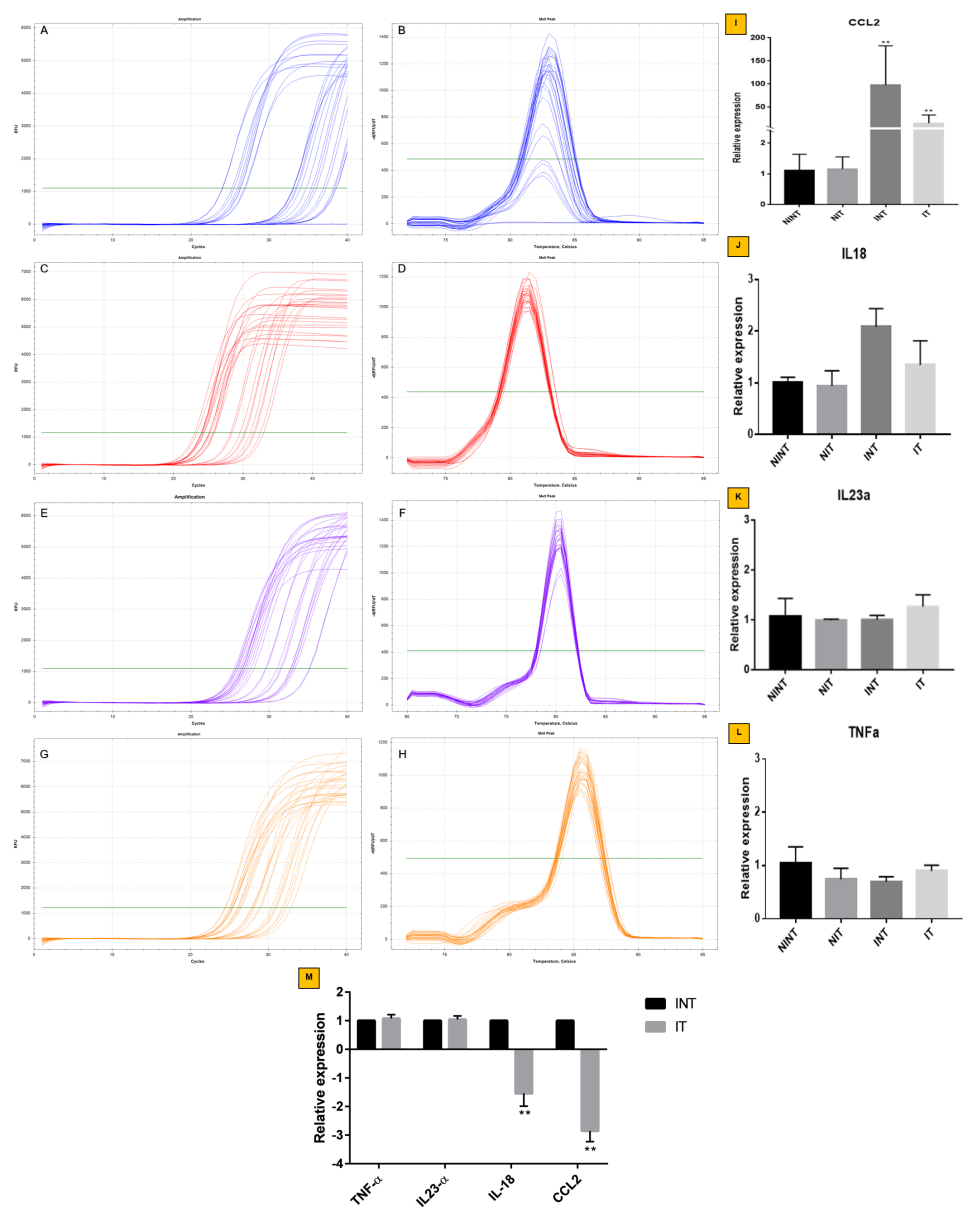
Relative mRNA expression of CCL2, IL-18, IL23α, and TNF-α in solid breast cancer after ECCT treatment. Amplification and melt peak chart of ( **A** and **B**) CCL2, ( **C** and **D**) IL18, ( **E** and **F**) IL23α and ( **G** and **H**) TNF-α. Relative expression of ( **I**) CCL2, ( **J**) IL-18, ( **K**) IL23α and ( **L**) TNF-α genes with NINT as a control group. The internal control of relative gene expression is GADPH gene. ( **M**) Relative expression of TNF-α, IL23α, IL-18, and CCL2 INT vs. IT. Error bar shows a standard deviation of three replications; **p<0.01.

The relative mRNA expression of CCL2, IL18, TNF-α, and IL23α genes can be seen in Figure 4. The appearance of CCL2(15.29 fold change), was significantly lower (p<0.01) on solid tumor tissues of the IT group than the INT group (97.72 fold change). This result was consistent with decreasing IL18 expression on tumor tissues of IT group (1.34 fold change) compared to the INT group (2.08 fold change). In contrast, the results of TNF-α and IL23α expression in both groups was not significantly different. However, TNF-α gene expression was up-regulated relatively and IL23α gene expression was down-regulated relatively with the internal control of GADPH gene expression. These results suggest that mRNA relative expression of CCL2 and IL18 were more down-regulated in tumor tissues exposed-ECCT (IT group) in comparison with the INT group. Whereas on the solid tumor tissues, the relative mRNA expression of both TNF-α and IL23α genes were similar.

## Discussion

DMBA-induced rat solid breast tumor has been studied in this study. Tumor interstitial fluid (TIF) formation could be affected by ECCT treatment, while other solid tumors not exposed to ECCT did not show a clearly visible effect. Although the substantial tumor size after ECCT exposure was not reduced, the solid tumor texture became soft and fluid ( Figure 1A and B). According to Wagner and Wig ^34^, the TIF contains stromal and immune cells able to produce inflammatory substances into the solid tumor environment. TIF is an essential source of tumor-specific proteins that can be used to examine the effect of therapy on the mechanism of tumor-promotion or inhibition. Here we connected the TIF phenomenon and the growth of tumor cells after treating rats with AC-electrical fields exposed ECCT. A previous study ^5^ using TTField (200 kHz) reported that this electric field treatment could influence not only tumor mitotic arrest and cell death, but also on cellular movement, infiltration and migration using *in vitro* and *in vivo* tests.

One of the critical modalities in cancer therapy is to minimize side effects resulting from the treatment. Our results ( Figure 2A and B) demonstrated that ECCT exposure on non-DMBA induced rats showed healthy mammary gland tissue, similar to those not exposed to ECCT (NIT and NINT groups, respectively). Normal lobules consisted of an epithelial cell layer in both treatments and there was no tissue damage, such as an atrophic duct or other tissue injuries. In the rat solid tumor tissues we observed an abundance of proliferative cells that expanded to most areas of mammary glands. The changing of cell composition on tumor nodule is not clearly defined. However, the condition of solid tumors treated with ECCT was better with than without exposure ( Figure 2C and D). Tumors treated with ECCT had a lower number (unseen data) of mitotic figures than the sham tumor treatment. Indeed, the apoptotic figures on tumor tissues with therapy were higher (unseen data) than non-therapy tissues ( Figure 2E and F). Necrotic tissue areas were found in both treatments, but were more frequent in ECCT exposed rat breast tumor tissues than non-exposed. A previous study on tumor cells and its microenvironment interaction suggested that molecules and intermediate signaling substances play a role in controlling cell infiltration, survival, and apoptosis, which occur at the same time. So far, these molecules could be used as molecular targets for anti-cancer therapy modality ^35^.

Alamsyah *et al.* ^1^ reported a decrease in solid tumor size after exposure with 100 kHz AC-EF ECCT. But in this study, we obtain different results, where the tumor size did not decrease using 150 kHz with a similar treatment method. Indeed, tumor size was not significantly increased. ECCT and TTFields are noninvasive cancer treatment modalities based on AC-EF with an intermediate frequency (100–200 kHz) and low intensity, however, they differ in noncontact and direct contact on skin, respectively, and wave type ^1, 3^. A recent study ^10^ reported that TTFields influence the activation of macrophage-specific immune responses through apoptotic bodies of dead tumor cells due to the agitation of cell mitosis by EF. These mechanisms should be further examined using molecular approaches, e.g. transcriptomics, to target proteins specifically affected by EF based cancer therapy.

Protein PCNA is frequently used as a cell proliferation marker. In this study, we evaluated the effect of ECCT treatment as an anti-proliferative agent using an intermediate frequency of 150 kHz. Previous studies examined the anti-proliferative effects using several types of cancer cell lines and measurement of tumor size *in vivo* ^1, 2^. In the current study, anti-proliferative activity can be seen in Figure 3A, B and I, which revealed that ECCT exposure enables inhibition of breast tumor cell proliferation (p<0.001). This result is consistent with previous findings ^1, 2^. In addition, Giladi *et al.* ^5^ exposed TTFields 150 kHz on non-small cell lung cancer cell lines and KLN205 squamous cell carcinoma in mice, which revealed inhibition of cancer cell viability and lung robust tumor growth due to the effect of AC-EF (TTFields) treatment. According to Ma *et al.* ^22^, DMBA promotes ErbB2-mediated carcinogenesis via genomic instability. In the present study ( Figure 3C, D and J), DMBA induced rats bearing solid breast tumors, which were exposed with ECCT treatment (IT), a significantly lower percentage of ErbB2 expressed cells were observed compared to the non-ECCT treatment (INT). Therefore, the present study supports previous results that suggested the anti-proliferative effect of AC-EF based tumor or cancer therapy (TTField and ECCT).

The most crucial evidence of cancer therapy is reducing tumor cell growth caused by cell death. Apoptosis is a necessary cell death process for inhibiting metastatic cancer ^36^. The present study shows that the percentage of breast tumor cells expressing caspase 3 (effector caspase) in the IT group is higher than in the INT group ( Figure 3E, F and K). It can be suggested that ECCT exposure disturbs cell mitosis toward cell death through apoptosis, autophagy or necrosis. However, this result indicates that apoptosis via caspase 3 might be a predominant mechanism of breast tumor cell death caused by ECCT-treatment. This result is consistent with the effect of reducing cell proliferation (using PCNA cell proliferation marker) in the IT group. Mujib *et al.* ^2^ suggested that p53 might be involved in apoptosis induction in several cancer cell lines via a caspase-dependent apoptosis mechanism after exposure to ECCT. The results shown by the present study need to be confirmed with other protein markers involved in the tumor cell death mechanism.

The clearance of cell death debris is the final stage of apoptosis. In this process, it involves phagocytic cells, such as macrophages. The current study ( Figure 3 G, H, and L) showed increasing expression of CD68 (a marker for macrophage and monocyte) on breast tumors exposed to ECCT (IT group), which was significantly higher than non-EF therapy (INT). Macrophages are multifunctional in solid tumor microenvironments, including macrophage polarization M1 and M2 which are involved in TAM and MAM functional directions ^7, 37^. Ni *et al.* ^38^ suggested that the CD68 positive macrophage is one of the tumor-infiltrating macrophages in non-metastatic breast cancer. Following the anti-proliferative effect of ECCT exposure, the up-regulation of classical CD68 macrophage marker may involve the expression of chemokine related macrophage polarization. This argument is supported by Park *et al*. ^10^, who reported that TTFs-induced inflammatory action is via the p38 MAPK/NF-kB signaling pathway and they believe that AC-EF based tumor therapy is a novel anticancer modality.

Solid tumor interstitial fluid is an excellent environment for communication of signaling substances among stromal, macrophages and tumor cells. In this study ( Figure 4), we evaluated the expression of CCL2, IL18, TNF-α, and IL23α, and whether they play a role in tumor progression or suppression. We demonstrated that the monocyte chemoattractant protein 1 (MCP-1) or CCL2 expression in solid breast tumor tissue with ECCT exposure (IT) is significantly lower (down-regulated) than without exposure (INT). There was no increase of relative mRNA CCL2 expression on ECCT exposure to the mammary gland of none DMBA induction rats (NIT) is shown in Figure 4I. This result supports a previous study that suggested that CCL2 has an essential role during breast cancer progression, through the induction of a systemic neutrophilic inflammatory cascade to facilitate metastasis ^39^. Moreover, Lavender *et al.* ^40^ reported that intra-tumoral CCL2 enables induction of breast tumor growth and/or metastases in breast cancer metastasis to the lung *in vivo*. Kirson *et al.* ^41^ reported that TTFields (100 kHz and 1-2 Vpp) exposure has a potential action to inhibit the development of lung cancer metastasis from primary cancer cells. Additionally, IL18 and IL10 act synergistically on angiogenesis progression ^8^. Following CCL2 expression, the present result demonstrated that IL18 expression is downregulated in breast tumor tissue exposed to ECCT treatment ( Figure 4C, D, J, and M). The high expression of IL18 in breast cancer enables promotion of cell proliferation and migration, and could be a biomarker candidate for prognosis in breast cancer patients ^42^. Indeed IL-18 expression leads to invasion and metastasis of breast cancer cells through PI3K-AKT/ ATF-2 signaling, and it might be regulated through NF-κB/NF-κB1 signaling in TAMs ^43^. Therefore, our result suggested that decreasing CCL2 and IL18 expression in the breast tumor microenvironment is most probably due to AC-EF treatment. We propose that there is a high correlation between the down-regulated expression of CCL2 and IL18 with the anti-proliferative effect of ECCT treatment. However, this argument should be further clarified by using other inflammatory markers of breast tumor progression.

In contrast with CCL2 and IL18 expression, in this study ( Figure 4M) the appearance of IL23α and TNF-α remains unchanged with the relative expression of both cytokines in normal mammary glands (NINT and NIT groups) and both solid breast tumor (INT and IT groups). A previous review ^44^ reported that a higher TNF-α expression is related to breast tumor progression which is elevated in stage II, III and IV but not in stage I. IL23 is involved in inflammation and angiogenesis in response to cytotoxic T-cell infiltration in the tumor microenvironment. Therefore, in this study the relationship between IL23α and TNF-α expression, and anti-proliferative effect of AC-EF based tumor therapy remain unclear.

## Conclusions

In conclusion, we propose that noncontact ECCT treatment could have an anti-proliferative effect on solid breast tumor via the down-regulated expression of CCL2 and IL18. This preclinical study may become a basis of consideration for a clinical trial toward the implementation of ECCT as a novel cancer therapy modality.

## Data availability

### Underlying data

Open Science Framework: CCL2 and IL18 expressions may associate with the anti-proliferative effect of noncontact electro capacitive cancer therapy *in vivo*. https://doi.org/10.17605/OSF.IO/EP7KT ^45^.

This project contains the following underlying data:

- IHC data
- Nodul data
- Raw images for IHC and hematoxylin figures
- Rat body weights during treatment
- qPCR data

Data are available under the terms of the Creative Commons Zero “No rights reserved” data waiver (CC0 1.0 Public domain dedication).

## References

[1] Alamsyah F Ajrina IN Dewi FNA: Antiproliferative Effect of Electric Fields on Breast Tumor Cells *In Vitro* and *In Vivo*. *Indones J Cancer Chemoprevent.* 2015;6(3):7 10.14499/indonesianjcanchemoprev6iss3pp71-77

[2] Mujib SA Alamsyah F Taruno WP: Cell Death and Induced p53 Expression in Oral Cancer, HeLa, and Bone Marrow Mesenchyme Cells under the Exposure to Noncontact Electric Fields. *Integr Med Int.* 2017;4(3–4):161–70. 10.1159/000485186

[3] Kirson ED Gurvich Z Schneiderman R: Disruption of cancer cell replication by alternating electric fields. *Cancer Res.* 2004;64(9):3288–95. 10.1158/0008-5472.CAN-04-0083 15126372

[4] Kirson ED Dbalý V Tovarys F: Alternating electric fields arrest cell proliferation in animal tumor models and human brain tumors. *Proc Natl Acad Sci U S A.* 2007;104(24):10152–7. 10.1073/pnas.0702916104 17551011 PMC1886002

[5] Giladi M Schneiderman RS Voloshin T: Mitotic Spindle Disruption by Alternating Electric Fields Leads to Improper Chromosome Segregation and Mitotic Catastrophe in Cancer Cells. *Sci Rep.* 2015;5:18046. 10.1038/srep18046 26658786 PMC4676010

[6] Timaner M Beyar-Katz O Shaked Y: Analysis of the Stromal Cellular Components of the Solid Tumor Microenvironment Using Flow Cytometry. *Curr Protoc Cell Biol.* 2016;70:19.8.1–19.18.12. 10.1002/0471143030.cb1918s70 26930555

[7] Kitamura T Qian BZ Soong D: CCL2-induced chemokine cascade promotes breast cancer metastasis by enhancing retention of metastasis-associated macrophages. *J Exp Med.* 2015;212(7):1043–59. 10.1084/jem.20141836 26056232 PMC4493415

[8] Kobori T Hamasaki S Kitaura A: Interleukin-18 Amplifies Macrophage Polarization and Morphological Alteration, Leading to Excessive Angiogenesis. *Front Immunol.* 2018;9:334. 10.3389/fimmu.2018.00334 29559970 PMC5845536

[9] Hoare JI Rajnicek AM McCaig CD: Electric fields are novel determinants of human macrophage functions. *J Leukoc Biol.* 2016;99(6):1141–51. 10.1189/jlb.3A0815-390R 26718542

[10] Park JI Song KH Jung SY: Tumor-Treating Fields Induce RAW264.7 Macrophage Activation Via NK-κB/MAPK Signaling Pathways. *Technol Cancer Res Treat.* 2019;18:1533033819868225. 10.1177/1533033819868225 31401938 PMC6691660

[11] Yang Y Cheon S Jung MK: Interleukin-18 enhances breast cancer cell migration via down-regulation of claudin-12 and induction of the p38 MAPK pathway. *Biochem Biophys Res Commun.* 2015;459(3):379–86. 10.1016/j.bbrc.2015.02.108 25727011

[12] Menegatti S Bianchi E Rogge L: Anti-TNF Therapy in Spondyloarthritis and Related Diseases, Impact on the Immune System and Prediction of Treatment Responses. *Front Immunol.* 2019;10:382. 10.3389/fimmu.2019.00382 30941119 PMC6434926

[13] Yu PF Huang Y Han YY: TNFα-activated mesenchymal stromal cells promote breast cancer metastasis by recruiting CXCR2 ^+^ neutrophils. *Oncogene.* 2017;36(4):482–490. 10.1038/onc.2016.217 27375023 PMC5290040

[14] Zhang H Liu K Xue Z: High-voltage pulsed electric field plus photodynamic therapy kills breast cancer cells by triggering apoptosis. *Am J Transl Res.* 2018;10(2):334–351. 29511429 PMC5835800

[15] Li K Wei L Huang Y: Leptin promotes breast cancer cell migration and invasion via IL-18 expression and secretion. *Int J Oncol.* 2016;48(6):2479–87. 10.3892/ijo.2016.3483 27082857

[16] Putra A Ridwan FB Putridewi AI: The role of tnf-α induced mscs on suppressive inflammation by increasing tgf-β and il-10. *Open Access Maced J Med Sci.* 2018;6(10):1779–1783. 10.3889/oamjms.2018.404 30455748 PMC6236029

[17] Hao NB Lu MH Fan YH: Macrophages in tumor microenvironments and the progression of tumors. *Clin Dev Immunol.* 2012;2012:948098. 10.1155/2012/948098 22778768 PMC3385963

[18] Langowski JL Kastelein RA Oft M: Swords into plowshares: IL-23 repurposes tumor immune surveillance. *Trends Immunol.* 2007;28(5):207–12. 10.1016/j.it.2007.03.006 17395538

[19] Lee C Jeong H Bae Y: Targeting of M2-like tumor-associated macrophages with a melittin-based pro-apoptotic peptide. *J Immunother Cancer.* 2019;7(1):1–14. 10.1186/s40425-019-0610-4 31174610 PMC6555931

[20] Fasoulakis Z Kolios G Papamanolis V: Interleukins Associated with Breast Cancer. *Cureus.* 2018;10(11):e3549. 10.7759/cureus.3549 30648081 PMC6324869

[21] Zimolag E Borowczyk-Michalowska J Kedracka-Krok S: Electric field as a potential directional cue in homing of bone marrow-derived mesenchymal stem cells to cutaneous wounds. *Biochim Biophys Acta Mol Cell Res.* 2017;1864(2):267–279. 10.1016/j.bbamcr.2016.11.011 27864076

[22] Ma Z Kim YM Howard EW: DMBA promotes ErbB2mediated carcinogenesis via ErbB2 and estrogen receptor pathway activation and genomic instability. *Oncol Rep.* 2018;40(3):1632–1640. 10.3892/or.2018.6545 30015966 PMC6072406

[23] Makiyah A: Effectiveness of Dose Concentration of Ethanol Extract of Iles-iles Tubers on Increasing Number of Macrophage Cells and Weight of Immune Organ Weight in White Rats Wistar Strain. *International Journal of Advances in Medical Sciences.* 2017;2(10):10 Reference Source

[24] Sulistyoningrum E Prasasti E Rachmani N: *Annona muricata* Leaves Extract Reduce Proliferative Indexes And Improve Histological Changes In Rat's Breast Cancer. *J appl pharm sci.* 2017;7(1):149–155. 10.7324/JAPS.2017.70120

[25] Firdaus AF Sobri I Ekowati H: Anti-Proliferative Activity of *Nigella sativa* Chloroform Extract on 7,12-Dimethylbenz[a]anthracene Induced Female Rats Splenocyte. *Indones J Cancer Chemoprevent.* 2012;3(1):351–7. 10.14499/indonesianjcanchemoprev3iss1pp351-357

[26] Cook DJ: Cellular Pathology.Butterworth-Heinemann.1998 Reference Source

[27] Fujimoto S Mochizuki K Shimada M: Variation in gene expression of inflammatory cytokines in leukocyte-derived cells of high-fat-diet-induced insulin-resistant rats. *Biosci Biotechnol Biochem.* 2008;72(10):2572–9. 10.1271/bbb.80259 18838815

[28] Poon K Abramova D Ho HT: Prenatal fat-rich diet exposure alters responses of embryonic neurons to the chemokine, CCL2, in the hypothalamus. *Neuroscience.* 2016;324:407–19. 10.1016/j.neuroscience.2016.03.017 26979053 PMC4838495

[29] Taki FA Abdel-Rahman AA Zhang B: A comprehensive approach to identify reliable reference gene candidates to investigate the link between alcoholism and endocrinology in Sprague-Dawley rats. *PLoS One.* 2014;9(5):e94311. 10.1371/journal.pone.0094311 24824616 PMC4019588

[30] Livak KJ Schmittgen TD: Analysis of relative gene expression data using real-time quantitative PCR and the 2(-Delta Delta C(T)) Method. *Methods.* 2001;25(4):402–8. 10.1006/meth.2001.1262 11846609

[31] Varghese F Bukhari AB Malhotra R: IHC Profiler: an open source plugin for the quantitative evaluation and automated scoring of immunohistochemistry images of human tissue samples. *PLoS One.* 2014;9(5):e96801. 10.1371/journal.pone.0096801 24802416 PMC4011881

[32] Pichler M Hutterer GC Chromecki TF: Histologic tumor necrosis is an independent prognostic indicator for clear cell and papillary renal cell carcinoma. *Am J Clin Pathol.* 2012;137(2):283–9. 10.1309/AJCPLBK9L9KDYQZP 22261455

[33] Denisov EV Skryabin NA Gerashchenko TS: Clinically relevant morphological structures in breast cancer represent transcriptionally distinct tumor cell populations with varied degrees of epithelial-mesenchymal transition and CD44 ^+^CD24 ^-^ stemness. *Oncotarget.* 2017;8(37):61163–61180. 10.18632/oncotarget.18022 28977854 PMC5617414

[34] Wagner M Wiig H: Tumor Interstitial Fluid Formation, Characterization, and Clinical Implications. *Front Oncol.* 2015;5:115. 10.3389/fonc.2015.00115 26075182 PMC4443729

[35] Ungefroren H Sebens S Seidl D: Interaction of tumor cells with the microenvironment. *Cell Commun Signal.* 2011;9:18. 10.1186/1478-811X-9-18 21914164 PMC3180438

[36] Su Z Yang Z Xu Y: Apoptosis, autophagy, necroptosis, and cancer metastasis. *Mol Cancer.* 2015;14:48. 10.1186/s12943-015-0321-5 25743109 PMC4343053

[37] Gerrick KY Gerrick ER Gupta A: Transcriptional profiling identifies novel regulators of macrophage polarization. *PLoS One.* 2018;13(12):e0208602. 10.1371/journal.pone.0208602 30532146 PMC6286176

[38] Ni C Yang L Xu Q: CD68- and CD163-positive tumor infiltrating macrophages in non-metastatic breast cancer: a retrospective study and meta-analysis. *J Cancer.* 2019;10(19):4463–4472. 10.7150/jca.33914 31528210 PMC6746141

[39] Kersten K Coffelt SB Hoogstraat M: Mammary tumor-derived CCL2 enhances pro-metastatic systemic inflammation through upregulation of IL1β in tumor-associated macrophages. *Oncoimmunology.* 2017;6(8):e1334744. 10.1080/2162402X.2017.1334744 28919995 PMC5593698

[40] Lavender N Yang J Chen SC: The Yin/Yan of CCL2: a minor role in neutrophil anti-tumor activity *in vitro* but a major role on the outgrowth of metastatic breast cancer lesions in the lung *in vivo*. *BMC cancer.* 2017;17(1):88. 10.1186/s12885-017-3074-2 28143493 PMC5286656

[41] Kirson ED Giladi M Gurvich Z: Alternating electric fields (TTFields) inhibit metastatic spread of solid tumors to the lungs. *Clin Exp Metastasis.* 2009;26(7):633–40. 10.1007/s10585-009-9262-y 19387848 PMC2776150

[42] Parikh R Kobawala T Trivedi T: Clinical utility of interleukin-18 in breast cancer patients: A pilot study. *Cancer Transl Med.* 2017;3(1):13–9. 10.4103/2395-3977.200855

[43] Li JH Fan WS Wang MM: Effects of mesenchymal stem cells on solid tumor metastasis in experimental cancer models: a systematic review and meta-analysis. *J Transl Med.* 2018;16(1):113. 10.1186/s12967-018-1484-9 29703232 PMC5924448

[44] Esquivel-Velázquez M Ostoa-Saloma P Palacios-Arreola MI: The role of cytokines in breast cancer development and progression. *J Interferon Cytokine Res.* 2015;35(1):1–16. 10.1089/jir.2014.0026 25068787 PMC4291218

[45] Pratiwi Rarastoeti: “CCL2 and IL18 Expressions May Associate with the Anti-proliferative Effect of Noncontact Electro Capacitive Cancer Therapy *in Vivo*.”OSF.2019 10.17605/OSF.IO/EP7KT PMC7348523 32695310

