# Supplementary material for: CCL2 and IL18 expressions may associate with the anti-proliferative effect of noncontact electro capacitive cancer therapy in vivo
Source: F1000Res. 2020 Jul 23;8:1770. Originally published 2019 Oct 17. [Version 2] doi: 10.12688/f1000research.20727.2 (PMC7348523; doi:10.12688/f1000research.20727.2)
Supplement: Peer review history

# Peer review: Reviewer response for version 2

**DOI:** 10.5256/f1000research.28071.r67858

**Published:** 2020-07-27

**Authors:** Agung Putra

**Affiliations:** 1 Stem Cell And Cancer Research (SCCR), School of Medicine, Sultan Agung Islamic University (UNISSULA), Semarang, Indonesia

Regarding the revised article, entitled "CCL2 and IL18 expressions may associate with the anti-proliferative effect of noncontact electro capacitive cancer therapy in vivo *", *all of the corrections have revised completely. The revisions provide all aspects that need to be improved, according to reviewer expectation and meet the acceptable scientific standards. Therefore, reviewer has accepted this article for indexing.

Is the work clearly and accurately presented and does it cite the current literature?

Yes

If applicable, is the statistical analysis and its interpretation appropriate?

Partly

Are all the source data underlying the results available to ensure full reproducibility?

Yes

Is the study design appropriate and is the work technically sound?

Yes

Are the conclusions drawn adequately supported by the results?

Yes

Are sufficient details of methods and analysis provided to allow replication by others?

Partly

Reviewer Expertise:

Mesenchymal Stem Cell, Immunology, Cancer

I confirm that I have read this submission and believe that I have an appropriate level of expertise to confirm that it is of an acceptable scientific standard.

# Peer review: Reviewer response for version 1

**DOI:** 10.5256/f1000research.22795.r65163

**Published:** 2020-07-09

**Authors:** Agung Putra

**Affiliations:** 1 Stem Cell And Cancer Research (SCCR), School of Medicine, Sultan Agung Islamic University (UNISSULA), Semarang, Indonesia

This manuscript addresses the effects of noncontact electro capacitive cancer therapy (ECCT) to down-regulate CCL2 and IL18 expressions in a breast cancer mouse model in which the end targets is inhibiting breast cancer cell proliferation. It is **an interesting topic** in efforts to eliminate cancer cells by elaboration between the anti-proliferative effect and activity of inflammatory cells following ECCT administration to breast cancer, due to the conventional therapy to reduce recurrence and breast cancer mortality **remains unclear**. On the other hand, the proinflammatory cells releasing proinflammatory cytokines such as IL18, TNF-a, IL23, and chemokine CCL2 also **play an essential role** in the development of breast tumors.

 In this paper, there are a number of issues regarding the methods and analysis that need to be clarified and addressed.

**Below are more specific comments by section**:

**Introduction:**

1. Minor corrections:

add a punctuation mark "comma" in this sentence:

- Stromal components, such as endothelial cells, myeloid derivate suppressor cells, and macrophages”

add “the” in this sentences:

- Furthermore, the evaluation of IL-23 suggested that this cytokine has… “

Add “-“ in this sentences:

- and growth by up-regulating matrix metalloproteinase….”

Add “a” in this sentences

- MCSs into a wound site and allow macrophages to be at a short distance to the wound…”

1. “Macrophages and tumor cells act as inflammatory cells that can be affected by chemical signals.” Tumor cells is not inflammatory cells.
2. So far, electric fields-based therapies need to be described in order to understand the modulation. The mechanism need to be investigated.
3. “TNF-α significantly induces breast cancer metastasis via TNFα-activated mesenchyme stem cells (MSCs) in lung.” However, under TNF-α stimulation at invitro model, the MSCs can also release IL-10 as anti-inflammatory cytokine that may be a beneficial for cancer growth inhibition.

Please read this article:

 Putra A, Ridwan FB, Putridewi AI, et al. The Role of TNF-α induced MSCs on Suppressive Inflammation by Increasing TGF-β and IL-10. Open Access Maced J Med Sci. 2018;6(10):1779-1783. Published 2018 Oct 4. doi:10.3889/oamjms.2018.404[ref-1].

1. “Tumor-associated macrophages (TAMs) may help tumor cell growth by releasing several pro-inflammatory cytokines, such as TNFα and IL23, which are expressed by the classically activated M1 macrophage.” Whereas, TAM is not classically activated M1 macrophage but the TAM is definitely an activated M2 macrophage.

Read this article:

 Lee C, Jeong H, Bae Y, et al. Targeting of M2-like tumor-associated macrophages with a melittin-based pro-apoptotic peptide. J Immunother Cancer. 2019;7(1):147. Published 2019 Jun 7. doi:10.1186/s40425-019-0610-4[ref-2].

1. A recent study demonstrated that TTFields therapy activates macrophage specific immune responses through the activation of several cytokines, such as NF-kB (nuclear factor-kB), TNF-α, and IL1-β….. NF-kB is not cytokine but the NF-Kb is the rapid-acting primary transcription factor for controlling cytokine production.

 
**Method:**

1. Minor corrections:

Add “this” in the sentence:

- The rat number for this experimental design was calculated for the minimal biological replication of rats…. “

Add “a” in the sentence:

- Tumor nodule diameters were measured every 2 days using a digital caliper ….”
- The sample paraffin blocks were sectioned with a microtome...”
- The stained samples were subsequently dehydrated using an upgraded level of...”

Add “-“ in the sentence:

 Solid tumor-bearing rats were exposed….

 Add “comma” in the sentence:

- using an upgraded level of alcohol, cleared in xylene, and lastly”

Remove “s” in the sentence:

- The INT and IT tumor tissues samples were then processed…”

Add “a” and “-“ in the sentence:

- In brief, the samples were deparaffinized using xylene and then rehydrated with a down-graded concentration of alcohol….”

1. Please clarify the experimental group. The experimental design of  this study used four rat groups with six biological replicates (n=6), however, the number of tissue sample required for replication is three tissues samples (n=3)...”

 
**Results**

1. The authors reported that ECCT-exposed rat breast tumor (IT) can induce the fluid bathing in tumors that associated with tumors size, in which the author's hypothesis is to inhibit breast tumor cell proliferation (anti-proliferation). Although, the results from the sham rat breast tumor group (INT) exhibited denser tissues and slower growth, however, the tumor volume of IT (treatment group) still exhibited larger than the sham (INT group), suggesting the result is on the contrary to the anti-proliferation. (Figure 1). Please clarify. 
2. The authors declared that the percentage of cells expressing CD68 protein in treatment groups (IT) were significantly higher than in the non-treatment groups (INT group) indicated that the macrophage polarized  into type-2, known as Tumor associated macrophage (TAM) that associated with the proliferation of cancer cell. Please clarify. 
3. On the other hand, in this study there was a decrease of CCL2 as the main chemokine of monocyte and macrophages, however the macrophage type-2 (CD 68 expression) was increased, Please clarify.

Is the work clearly and accurately presented and does it cite the current literature?

Yes

If applicable, is the statistical analysis and its interpretation appropriate?

Partly

Are all the source data underlying the results available to ensure full reproducibility?

Yes

Is the study design appropriate and is the work technically sound?

Yes

Are the conclusions drawn adequately supported by the results?

Yes

Are sufficient details of methods and analysis provided to allow replication by others?

Partly

Reviewer Expertise:

Mesenchymal Stem Cell, Immunology, Cancer

I confirm that I have read this submission and believe that I have an appropriate level of expertise to confirm that it is of an acceptable scientific standard, however I have significant reservations, as outlined above.

# Response

**Published:** 2020-07-14

**Authors:** Rarastoeti Pratiwi

**Affiliations:** Universitas Gadjah Mada, Indonesia

**External Reviewer (Dr. A. Putra):**

 APPROVED WITH RESERVATIONS

 This manuscript addresses the effects of noncontact electro capacitive cancer therapy (ECCT) to down-regulate CCL2 and IL18 expressions in a breast cancer mouse model in which the end targets is inhibiting breast cancer cell proliferation. It is **an interesting topic** in efforts to eliminate cancer cells by elaboration between the anti-proliferative effect and activity of inflammatory cells following ECCT administration to breast cancer, due to the conventional therapy to reduce recurrence and breast cancer mortality **remains unclear**. On the other hand, the proinflammatory cells releasing proinflammatory cytokines such as IL18, TNF-a, IL23, and chemokine CCL2 also **play an essential role** in the development of breast tumors.

 In this paper, there are a number of issues regarding the methods and analysis that need to be clarified and addressed.

**Below are more specific comments by section**:

**Introduction:**

1. Minor corrections:

add a punctuation mark "comma" in this sentence:

- Stromal components, such as endothelial cells, myeloid derivate suppressor cells, and macrophages”

add “the” in this sentences:

- Furthermore, the evaluation of IL-23 suggested that this cytokine has… “
- Furthermore, evaluation of IL-23 suggested

Add “-“ in this sentences:

- and growth by up-regulating matrix metalloproteinase….”
- and growth by up regulating

Add “a” in this sentences

- MCSs into a wound site and allow macrophages to be at a short distance to the wound…”
- MCSs into a wound site and allow macrophages to be at short distance to the wound.

1. “Macrophages and tumor cells act as inflammatory cells that can be affected by chemical signals.” Tumor cells is not inflammatory cells.

**User comment:**

 All typo corrections have been done.

 So far, electric fields-based therapies need to be described in order to understand the modulation. The mechanism need to be investigated.

**User comment:**

 Revision has been done. In the new version article has been added “The mechanism needs to be investigated”.

1. “TNF-α significantly induces breast cancer metastasis via TNFα-activated mesenchyme stem cells (MSCs) in lung.” However, under TNF-α stimulation at invitro model, the MSCs can also release IL-10 as anti-inflammatory cytokine that may be a beneficial for cancer growth inhibition.

Please read this article:

 Putra A, Ridwan FB, Putridewi AI, et al. The Role of TNF-α induced MSCs on Suppressive Inflammation by Increasing TGF-β and IL-10. Open Access Maced J Med Sci. 2018;6(10):1779-1783. Published 2018 Oct 4. doi:10.3889/oamjms.2018.404[ref-1].

**User comment:**

 In the new version, a revision has been done by added a new reference: reference no 16.

  “Tumor-associated macrophages (TAMs) may help tumor cell growth by releasing several pro-inflammatory cytokines, such as TNFα and IL23, which are expressed by the classically activated M1 macrophage.” Whereas, TAM is not classically activated M1 macrophage but the TAM is definitely an activated M2 macrophage.

 Read this article:

 Lee C, Jeong H, Bae Y, et al. Targeting of M2-like tumor-associated macrophages with a melittin-based pro-apoptotic peptide. J Immunother Cancer. 2019;7(1):147. Published 2019 Jun 7. doi:10.1186/s40425-019-0610-4[ref-2].

**User comment:**

 In the new version, a revision has been done by added a new reference: reference no 19.

 3. A recent study demonstrated that TTFields therapy activates macrophage specific immune responses through the activation of several cytokines, such as NF-kB (nuclear factor-kB), TNF-α, and IL1-β….. NF-kB is not cytokine but the NF-Kb is the rapid-acting primary transcription factor for controlling cytokine production.

**User comment:**

 Revision has been done by deleting: “NF-kB (nuclear factor-kB),”

**Method:**

1. Minor corrections:

Add “this” in the sentence:

- The rat number for this experimental design was calculated for the minimal biological replication of rats…. “

Add “a” in the sentence:

- Tumor nodule diameters were measured every 2 days using a digital caliper ….”
- The sample paraffin blocks were sectioned with a microtome...”
- The stained samples were subsequently dehydrated using an upgraded level of...”

The stained samples were subsequently dehydrated using upgraded level

 Add “-“ in the sentence:

 Solid tumor-bearing rats were exposed….

 Add “comma” in the sentence:

- using an upgraded level of alcohol, cleared in xylene, and lastly”

using an upgraded level of alcohol, cleared in xylene and, lastly,

 Remove “s” in the sentence:

- The INT and IT tumor tissues samples were then processed…”

Add “a” and “-“ in the sentence:

- In brief, the samples were deparaffinized using xylene and then rehydrated with a down-graded concentration of alcohol….”

**User comment:**

 In the new version, all these typo corrections have been done.

1. Please clarify the experimental group. The experimental design of  this study used four rat groups with six biological replicates (n=6), however, the number of tissue sample required for replication is three tissues samples (n=3)...”

**User comment:**

 Revision:

 In the new version, the (n=3) have been deleted in the explanations of:”

 ·         - NINT group: non-DMBA-induced rats, non-therapy exposure (n=3)

 ·         - NIT group: non-DMBA-induced rats, therapy exposure (n=3)

 ·         - INT: DMBA-induced rats, non-therapy treatment (n=3)

 ·         - IT: DMBA-induced rate, therapy exposure (n=3)”, because it could confuse.

      

 In the new version, the replication of rat (n=6) and three tissues sample per treatment group (n=3) have been added in the text. So, the NINT, NIT, INT and IT just to explain the treatment for each group (without (n=3).

 Clarification:

 As I described in the manuscript:  “The rat number for this experimental design was calculated for the minimal biological replication of rats, with four treatment groups according to the Federer Formula ^2^ ^3^ “ and this number also follow the ethical procedure for using the minimal animals. However, we only using a minimal number of tissue samples replication requirement for statistical analysis in random sampling method. Three tissues samples per treatment group (4 groups) was used for a biological replication requirement. Each sample was triple sections per tissue sample for histopathologic scoring requirements (to gain 50 fields of view) and twice for qRT-PCR analysis. However, we used six rats (n=6) per group to observe rat body weights (secondary data)

**Results**

 1. The authors reported that ECCT-exposed rat breast tumor (IT) can induce the fluid bathing in tumors that associated with tumors size, in which the author's hypothesis is to inhibit breast tumor cell proliferation (anti-proliferation). Although, the results from the sham rat breast tumor group (INT) exhibited denser tissues and slower growth, however, the tumor volume of IT (treatment group) still exhibited larger than the sham (INT group), suggesting the result is on the contrary to the anti-proliferation. (Figure 1). Please clarify.

**User comment:**

 Clarification:

 The larger size of tumor nodules in IT groups due to the measurement using a digital caliper. In that case, we measured the nodule volume included the inflammation fluids, instead of tumor tissues. However, from the histological observation, the results showed that the number of proliferation cells in IT groups significantly lower than in INT group. So, from this result we suggest that the tumor interstitial fluid contains stromal and immune cells could increase the tumor nodule volume. Further, we observed the proliferation of the tumor cells (based on PCNA marker of cells), and the role of macrophage (based on CD68 marker of monocyte and macrophage) to eliminate the tumor cells which are failure to divided.

 2. The authors declared that the percentage of cells expressing CD68 protein in treatment groups (IT) were significantly higher than in the non-treatment groups (INT group) indicated that the macrophage polarized  into type-2, known as Tumor associated macrophage (TAM) that associated with the proliferation of cancer cell. Please clarify. 

**User comment:**

 Revision:

 In the new revision, the words “a classical”, in the sentence: ” ... expression of CD68 (a classical macrophage marker)..” has been deleted and edited: “…expression of CD68 (a marker for macrophage and monocyte)

 Clarification:

 CD68 is the marker of macrophage and monocyte (see the revision obove), so these macrophages could be M1 and M2 (TAM).

 3. On the other hand, in this study there was a decrease of CCL2 as the main chemokine of monocyte and macrophages, however the macrophage type-2 (CD 68 expression) was increased, Please clarify.

**User comment:**

 Clarificasion:

 CD68 can be expressed either in monocyte and macrophage (M1 and M2). However, CCL2 level correlated with TAM abundance and TAM is M2 Macrophage. The results show that the increase of macrophage CD68 (could be M1 and M2 macrophages) in IT group, but the expression of CCL2 in this group is decrease.

- **Is the work clearly and accurately presented and does it cite the current literature?**

Yes

- **Is the study design appropriate and is the work technically sound?**

Yes

- **Are sufficient details of methods and analysis provided to allow replication by others?**

Partly

- **If applicable, is the statistical analysis and its interpretation appropriate?**

Partly

- **Are all the source data underlying the results available to ensure full reproducibility?**

Yes

- **Are the conclusions drawn adequately supported by the results?**

Yes

 Competing Interests

*No competing interests were disclosed.*

 Reviewer Expertise

 Mesenchymal Stem Cell, Immunology, Cancer

**I confirm that I have read this submission and believe that I have an appropriate level of expertise to confirm that it is of an acceptable scientific standard, however I have significant reservations, as outlined above.**

# Peer review: Reviewer response for version 1

**DOI:** 10.5256/f1000research.22795.r59978

**Published:** 2020-03-05

**Authors:** Yoram Palti

**Affiliations:** 1 Novocure, Haifa, Israel

The manuscript by Antara and colleagues reports on the use of noncontact ECCT treatment to down-regulated expression of CCL2 and IL18 and therefore inhibit breast cancer cell proliferation.

**Comments:**

1. The authors claim that the fluid bathing in breast tumors from ECCT-exposed IT treated rats may affect the tumors size, however, no specific analysis was done to support such claim. For instance, a single cell suspension of whole tumor followed by cell count could suffice. This data is crucial In order to support the claim that ECCT treatment is anti-proliferative, as tumor volume measurements show otherwise.
2. The authors should also elaborate on:

- The impact of reduction in CCL2 and phenotype skewing in tumor macrophages.
- The discrepancy between CCL2 levels and CD68 infiltration.
- The discrepancy between past publications showing activation of the MAPK/NF-kB signaling pathway following AC-EF based tumor therapy (suggested as an example that has been used by the authors to support some of the conclusions in the current study) and the downregulation of IL18 in the current study.

Is the work clearly and accurately presented and does it cite the current literature?

Yes

If applicable, is the statistical analysis and its interpretation appropriate?

I cannot comment. A qualified statistician is required.

Are all the source data underlying the results available to ensure full reproducibility?

Partly

Is the study design appropriate and is the work technically sound?

Partly

Are the conclusions drawn adequately supported by the results?

No

Are sufficient details of methods and analysis provided to allow replication by others?

Yes

Reviewer Expertise:

NA

I confirm that I have read this submission and believe that I have an appropriate level of expertise to confirm that it is of an acceptable scientific standard, however I have significant reservations, as outlined above.

# Peer review: Reviewer response for version 1

**DOI:** 10.5256/f1000research.22795.r55336

**Published:** 2019-10-23

**Authors:** Richard Luke Daniels

**Affiliations:** 1 Department of Biology, The College of Idaho, Caldwell, ID, USA

This paper describes the effects of intermediate frequency (100-300 kHz) alternative electric fields in a mouse model of breast cancer. Specifically, the authors examined expression (by immunohistochemistry and/or qPCR) of several genes. These were immune factors known to be associated with tumor cell proliferation and angiogenesis (CCL2 and IL18), a marker of cell proliferation (PCNA), a growth factor receptor (ErbB2), a marker of leukocyte differentiation (CD68) several other immune factors (IL18, IL23-alpha, TNF-alpha), and a marker of apoptosis (caspase-3). Changes were seen in gene expression consistent with decreased cell proliferation and increased cell death, though pro-inflammatory changes were not seen. These results provide evidence that supports a biological effect of alternating electric fields on cell proliferation, and it is this reviewer’s opinion that publication is warranted. There are a few minor modifications that I would suggest making before indexing this manuscript, which center around experiments described in figures 2-4.

**Minor English language corrections:**

 In some instances, “none” should be changed to “non” throughout the document, as in “non DMBA-induced” (this is sometimes correctly stated, for example the animal methods section is correct).

 In the conclusion, change “purpose” to “propose”

**Methods:**

 It would be beneficial to give more specifics about the protein quantification done using ImageJ. Was the scoring automated in anyway? What was the process or procedure used for determining whether a cell was positive for a given marker?

**Figure 2:**

 The figure supports what is described in the text; however no quantification is given regarding the stated observations regarding the cell composition of the tumor and the structural changes with and without treatment. I do not consider this to be essential for indexing, however it would strengthen the conclusions drawn from this experiment if these results could be quantified.

**Figure 3:**

 The figure supports what is described in the text. See comments related to the scoring method above. In the statistics, it is a bit unclear as to what was counted as an individual n. Was each slide counted as a single n? Was each image (50 images / slide)? It would be helpful for context to describe in a bit more detail either in these results or the methods section what was counted as an individual experiment for interpretation of the statistical comparisons.

**Figure 4:**

 This figure supports what is described in the text. The graphs would benefit from changing the Y-axes to be consistently labeled with the same amount of significant figures. Also – if the CCL2 expression is 100-fold different than NIT, etc., it might be beneficial to point this out in the discussion, as this increase is substantially more than other differences seen that are 2-fold different, for example.

Is the work clearly and accurately presented and does it cite the current literature?

Yes

If applicable, is the statistical analysis and its interpretation appropriate?

Partly

Are all the source data underlying the results available to ensure full reproducibility?

No source data required

Is the study design appropriate and is the work technically sound?

Yes

Are the conclusions drawn adequately supported by the results?

Partly

Are sufficient details of methods and analysis provided to allow replication by others?

Partly

Reviewer Expertise:

Cell physiology, Neuroscience, Cell signaling

I confirm that I have read this submission and believe that I have an appropriate level of expertise to confirm that it is of an acceptable scientific standard.

# Response

**Published:** 2020-07-14

**Authors:** Rarastoeti Pratiwi

**Affiliations:** Universitas Gadjah Mada, Indonesia

**External Reviewer (Dr. R. L. Daniels):**

 This paper describes the effects of intermediate frequency (100-300 kHz) alternative electric fields in a mouse model of breast cancer. Specifically, the authors examined expression (by immunohistochemistry and/or qPCR) of several genes. These were immune factors known to be associated with tumor cell proliferation and angiogenesis (CCL2 and IL18), a marker of cell proliferation (PCNA), a growth factor receptor (ErbB2), a marker of leukocyte differentiation (CD68) several other immune factors (IL18, IL23-alpha, TNF-alpha), and a marker of apoptosis (caspase-3). Changes were seen in gene expression consistent with decreased cell proliferation and increased cell death, though pro-inflammatory changes were not seen. These results provide evidence that supports a biological effect of alternating electric fields on cell proliferation, and it is this reviewer’s opinion that publication is warranted. There are a few minor modifications that I would suggest making before indexing this manuscript, which center around experiments described in figures 2-4.

**Minor English language corrections:**

 In some instances, “none” should be changed to “non” throughout the document, as in “non DMBA-induced” (this is sometimes correctly stated, for example the animal methods section is correct).

 In the conclusion, change “purpose” to “propose”

**User comment:**

 All typo corrections have been done

**Methods:**

 It would be beneficial to give more specifics about the protein quantification done using ImageJ. Was the scoring automated in anyway? What was the process or procedure used for determining whether a cell was positive for a given marker?

**User comment**

 In the new version article has been revised: "Data analysis for IHC was scored automatically using color deconvolution and computerized pixel profiling by IHC Profiler plugin on ImageJ version 1.51 software ^29"^

**Figure 2:**

 The figure supports what is described in the text; however no quantification is given regarding the stated observations regarding the cell composition of the tumor and the structural changes with and without treatment. I do not consider this to be essential for indexing, however it would strengthen the conclusions drawn from this experiment if these results could be quantified".

**User comment:**

 In the new version article has been revised:"The tumor nodule is not a complex tissue, usually consist of connective and alveolar tissues, therefore the changing of cell composition is not clearly defined".

**Figure 3:**

 The figure supports what is described in the text. See comments related to the scoring method above. In the statistics, it is a bit unclear as to what was counted as an individual n. Was each slide counted as a single n? Was each image (50 images / slide)? It would be helpful for context to describe in a bit more detail either in these results or the methods section what was counted as an individual experiment for interpretation of the statistical comparisons.

**User comment**

 In the new version article has been revised:"The random 50 fields of view on IHC slides of each treatment were observed under Leica ICC50 E at 0.5 µm/pixel resolution".

**Figure 4:**

 This figure supports what is described in the text. The graphs would benefit from changing the Y-axes to be consistently labeled with the same amount of significant figures. Also – if the CCL2 expression is 100-fold different than NIT, etc., it might be beneficial to point this out in the discussion, as this increase is substantially more than other differences seen that are 2-fold different, for example.

**User Comment**

 A revision has been done in the new version article:"The appearance of CCL2(15.29 fold change), was significantly lower (p<0.01) on solid tumor tissues of the IT group than the INT group (97.72 fold change). This result was consistent with decreasing IL18 expression on tumor tissues of IT group (1.34 fold change) compared to the INT group (2.08 fold change)". Figure 4.I. has been edited for the scale of expression relative (fold change).

- **Is the work clearly and accurately presented and does it cite the current literature?**

Yes

- **Is the study design appropriate and is the work technically sound?**

Yes

- **Are sufficient details of methods and analysis provided to allow replication by others?**

Partly

- **If applicable, is the statistical analysis and its interpretation appropriate?**

Partly

- **Are all the source data underlying the results available to ensure full reproducibility?**

No source data required

- **Are the conclusions drawn adequately supported by the results?**

Partly

 Competing Interests

*No competing interests were disclosed.*

 Reviewer Expertise

 Cell physiology, Neuroscience, Cell signaling
